# Huangkui Lianchang Decoction Ameliorates DSS-Induced Ulcerative Colitis in Mice by Inhibiting the NF-kappaB Signaling Pathway

**DOI:** 10.1155/2019/1040847

**Published:** 2019-04-10

**Authors:** Zongqi He, Qing Zhou, Ke Wen, Bensheng Wu, Xueliang Sun, Xiaopeng Wang, Yugen Chen

**Affiliations:** ^1^Department of Colorectal Surgery, Affiliated Hospital of Nanjing University of Chinese Medicine, Nanjing 210046, China; ^2^Department of Colorectal Surgery, Suzhou Hospital Affiliated with Nanjing University of Chinese Medicine, Suzhou 215009, China

## Abstract

**Background:**

The nuclear factor kappa beta (NF-*κ*B) signaling pathway plays an important role in ulcerative colitis (UC). Huangkui Lianchang decoction (HLD) is an effective traditional Chinese medicinal compound used in the treatment of UC. HLD has good effects in the clinic, but the mechanism by which HLD acts is unclear. This study aims to reveal the exact molecular mechanism of HLD in the treatment of UC.

**Methods:**

Mouse ulcerative colitis was induced by dextran sulfate sodium (DSS) and treated with HLD. Intestinal damage was assessed by disease activity index (DAI), colon macroscopic lesion scores, and histological scores. Interleukin (IL)-6, tumor necrosis factor (TNF)-*α*, and IL-1*β* were detected in colon tissue using ELISA. Myeloperoxidase (MPO) and superoxide dismutase (SOD) activities in the colonic mucosa were measured. The levels of IL-6, inducible nitric oxide synthase (iNOS), and cyclooxygenase-2 (COX-2) in the colon were determined by real-time quantitative polymerase chain reaction (qPCR). The expression of NF-*κ*B, I*κ*B*α*, and p-I*κ*B*α* in the colon was measured by Western blot.

**Results:**

After treatment with HLD, the DAI scores, macroscopic lesion scores, and histological scores decreased, and the levels of inflammatory cytokines related to the NF-*κ*B signaling pathway, such as IL-6, TNF-*α*, and IL-1*β*, as well as those of iNOS and COX-2, were reduced; at the same time, colonic pathological damage was alleviated, and the MPO and SOD activities decreased. Western blot confirmed that HLD can inhibit the NF-*κ*B signaling pathway in DSS-induced ulcerative colitis.

**Conclusion:**

HLD can alleviate the inflammation caused by ulcerative colitis. In particular, high doses of HLD can significantly alleviate intestinal inflammation and have comparable efficacy to Mesalazine. We propose that the anti-inflammatory activity of HLD on DSS-induced colitis in mice may involve the inhibition of the NF-*κ*B pathway.

## 1. Introduction

Inflammatory bowel disease refers to nonspecific intestinal inflammatory diseases of unknown etiology, including ulcerative colitis and Crohn's disease [1]. Ulcerative colitis is more common in the West, but in recent years, the incidence of UC has been rising constantly, especially in Asia, and the disease seriously endangers people's health [2–4]. Active ulcerative colitis mainly manifests as abdominal pain, diarrhea, and mucus pus and blood in stool while the pathogenesis of UC is still unclear, which may be related to genetic factors, environmental factors, infection, dysbacteriosis, and immune factors [5]. Among those factors, the immune component of UC pathogenesis is the most widely accepted, and it is also a research hotspot [6].

Many studies have found that NF-*κ*B plays an important role in inflammation [1, 7, 8]. Under normal physiological conditions, the p65/p50 NF-*κ*B heterodimer remains inactive in complex with a kappa B inhibitor (I*κ*B*α*) [7]. NF-*κ*B activity is triggered through the phosphorylation and degradation of I*κ*B*α* in response to inflammatory stimuli and subsequent translocation of NF-*κ*B to the nucleus where NF-*κ*B controls target gene expression and activates the proinflammatory cytokines IL-6, IL-1*β*, and TNF-*α* as well as proinflammatory substances such as iNOS and COX-2, which induce inflammation [8]. Although great progress has been made in elucidating the pathogenesis of ulcerative colitis, further research is needed.

Because the cause of UC is unknown, current treatments relieve symptoms but do not cure UC. Thus far, the medications used to treat UC mainly include aminosalicylic acid, hormones, immunosuppressants, and anti-TNF-*α* drugs, which can reduce inflammation and weaken the expression of inflammatory factors [9]. However, the above medications do not cure UC and cause adverse effects, such as abnormalities in the blood, abnormal liver and kidney function, serious infections, osteoporosis and metabolic disorders, in both short- and long-term treatment courses [10–12]. Additionally, the side effects from long-term medication use reduce the quality of life and satisfaction of patients [4, 11]. Therefore, there is an urgent need to find drugs with good curative effects and few side effects.

The use of traditional, complementary, and alternative medicines has attracted special attention among some communities because such treatments have fewer adverse effects compared to standard medications [13]. An increasing number of Chinese medicines have been proven to possess good anti-inflammatory, antitumor, and immune regulatory effects [14, 15]. Huangkui Lianchang decoction (HLD) is a traditional Chinese medicinal cocktail used to treat ulcerative colitis. The use of HLD is based on the experience of Professor Chen Yugen of Nanjing University of Chinese Medicine. HLD is composed of 6 traditional Chinese medicines: the main medicine is Abelmoschus manihot (L.) Medik (huang shu kui hua), which is combined with Euphorbia humifusa Willd (di jin cao), Pteris multifida Poir (feng wei cao), Lithospermum erythrorhizon Siebold & Zucc (zi cao), Rubia cordifolia L. (qian cao), and Rhus chinensis Mill (wu bei zi). The proposed function of HLD is clearing heat and dampness, activating blood, promoting Qi, protecting the intestine and detoxifying. In practice, mild to moderate E1 and E2 type UC is treated with HLD enema alone, while severe or E3 type UC is treated with HLD enema combined with oral medication. We have achieved good results in the clinical application of Huangkui Lianchang decoction for the treatment of mild or moderate UC, but the molecular mechanism of HLD is unclear, and the optimal dose is unknown. Yang et al. found that total flavone of Abelmoschus manihot (the extract of Abelmoschus manihot(L.) Medik) can inhibit intestinal fibrosis in Crohn's disease via interfering TGF-*β*1 signaling [16]. Qiu et al. found that total flavone of Abelmoschus manihot can improve oxidative stress and reduce TNF-*α* and IL-1*β* in the liver of mice through the Nrf2 pathway [17]. Li et al. also found the antioxidant activity of total flavone of Abelmoschus manihot [18]. Researchers from South Korea reported that Euphorbia humifusa Willd has anti-inflammatory properties and its extract can inhibit nitric oxide and TNF-*α*[19]. Another study from South Korea found that Euphorbia humifusa Willd can inhibit the invasion and metastasis of early breast cancer by inhibiting NF-*κ*B [20]. Similarly, one study reported that shikonin (the extract of Lithospermum erythrorhizon Siebold & Zucc) has anti-inflammatory activities [21]. The basic studies above have only studied one of the components (or its extract) in HLD. However, no further studies have been conducted on compound HLD.

To this end, we designed this experiment to determine the therapeutic effect and efficacy-dose relationship of HLD on DSS-induced UC. We hypothesized that Huangkui Lianchang decoction alleviates UC through the NF-*κ*B pathway.

## 2. Materials and Methods

### 2.1. Animals and Medicines

Thirty-six SPF male Balb/c mice were purchased from the Animal Experimental Center of Suzhou University and weighed 13.6 g to 16.8 g. Mice were housed in clean laboratory animal rooms at a temperature of 20-22°C with a 12 hour light/dark cycle. Prior to experimentation, Balb/c mice were given free access to food and water and were allowed to adapt to the environment for one week. Abelmoschus manihot (L.) Medik (huang shu kui hua), Euphorbia humifusa Willd (di jin cao), Pteris multifida Poir (feng wei cao), Lithospermum erythrorhizon Siebold & Zucc (zi cao), Rubia cordifolia L. (qian cao), and Rhus chinensis Mill (wu bei zi) were mixed in a ratio of 6:6:6:3:3:1, and they were purchased from Suzhou Tianling Chinese Medicine Pieces Co., Ltd. All herbs were used in accordance with the Chinese Pharmacopoeia standards identified by the Pharmacy Department of Suzhou Traditional Chinese Medicine Hospital. After the herbs were washed with water, they were soaked in distilled water equivalent to 5 times the amount of the medicine for 60 minutes, boiled for 30 minutes, and filtered; the dregs were added to 3 times the amount of distilled water, boiled for another 20 minutes, and filtered [22]. The two decoctions were mixed and diluted to 1.92 g of crude drug/mL of drug solution in a water bath for use. Mesalazine sustained-release granules (Etiasa, 0.5 g/sachet, Shanghai Ethypharm Pharmaceutical Co., Ltd.) were used as standard therapeutic drug [23]. All other chemicals and solvents were analytical-grade commercial products.

### 2.2. Modeling and Group Administration

This animal experiment complyed with the ARRIVE guidelines and was carried out in accordance with the National Institutes of Health guide for the care and use of laboratory animals. This experiment has been approved by the Animal Experimental Ethics Committee of Suzhou University. On the first day of the experiment, each mouse was weighed and recorded twice, and the average value was taken as the initial body weight. The UC model was induced by the DSS method [24–27]. Briefly, 15 g DSS powder (Shanghai Xusheng Biotechnology Co., Ltd., China) was dissolved in 500 mL sterile water to make a 3% DSS solution (molecular weight 36,000-50,000 Da).

Six mice were left untreated and used as a Control group, while the remaining 30 mice were subjected to DSS treatment. At the start of the experiment, 3% DSS solution (5 ml per mouse per day) was used instead of drinking water. The DSS solution was replaced on the 3rd and 5th days. On 8th day, the remaining DSS solution was replaced with sterile water. After successful DSS treatment, mice were randomly divided into 5 groups (DSS, Mesalazine, HLD-L, HLD-M, HLD-H) with 6 mice in each group. According to the “Methodology of Pharmacological Research of Traditional Chinese Medicine”, the bioavailability of the HLD-M group was 18.85 g/kg; doses 1/2 and 2 times of the bioavailability of the HLD-M group were used for the HLD-L group (9.425 g/kg) and HLD-H group (37.70 g/kg), respectively. Mesalazine group: the dosage of adult acute UC Mesalazine was 4 g/d, and it was administered in a single dose of 0.52 g/kg in mice. The medicine administered to the HLD-L, HLD-M, and HLD-H groups was diluted with distilled water such that each group of mice received an equal volume of drug enema. The Control group and the DSS group were given 1 mL of distilled water per day. The Control, DSS, HLD-L, HLD-M, HLD-H, and Mesalazine group enemas were all administered starting on the 2nd day after successful modeling and continuing for 14 consecutive days.

### 2.3. Disease Activity Index (DAI) Scores

The body weight, stool consistency, and blood in the stool were observed and recorded during the experiment, and DAI was calculated accordingly as shown in Table 1 [28].

### 2.4. Colonic Macroscopic Lesion Evaluation

After the last enema administration, all mice were fasted for 24 hours and then sacrificed after anesthetized by chloral hydrate. The colon from anus to the ileocecum of all mice was removed and was dissected along the mesenteric longitudinal line to observe inflammation of the colonic mucosa. The criteria for evaluating colonic macroscopic lesions were the following: diarrhea (0=no, 1=yes), ulcer (0 = no injury, 1 = congestion, no ulcer, 2 = ulcer but no congestion or thickening of the intestinal wall, 3 =1 ulcer with inflammation, 4 = ≥ 2 ulcers/inflammation, 5 = 1 major lesion along the longitudinal axis of the colon is >1 cm, and 6-10=1 lesion is >2 cm; for scores from 6-10, the points increase by 1 for every 1 cm increase in lesion size) and adhesion (0=no adhesion, 1=mild adhesion, where the colon can be easily separated from the adhesion tissue, 2= severe adhesion) [29, 30].

### 2.5. Histological Score

Specimens with obvious inflammation/ulcer or specimens 15 cm away from the anus (if a mouse had no obvious lesion) were chosen for histological examination. Next, specimens were embedded in paraffin, sectioned, HE stained, and observed for intestinal histological changes. Histological score was assessed by a professional pathologist who was blinded to the study. Histological scores were assigned in parallel as follows: 0 = normal tissue, 1 = superficial epithelial damage, 2 = focal ulcer confined to the mucosa, 3 = focal transmural inflammation and ulceration, 4 = extensive transmural ulcer and inflammation, normal mucosa between lesions, 5 = extensive flaky transmural ulcers and inflammation [28]. Part of each colon was taken and fixed in 10% formalin for other tests.

### 2.6. Measurement of IL-6, TNF-*α*, and IL-1*β* in Colon Tissue by ELISA

The levels of IL-6, TNF-*α*, and IL-1*β* were measured in colon tissue by a RAYTO RT-6000 ELISA detector following the standard detection procedure. The procedure is as follows: (1) added sample: added 0.1 ml of the sample to the reaction well, incubated at 37°C for 1 hour; (2) washed: washed the sample with PBST for five times, 30 seconds each time; (3) added enzyme-labeled antibody: 0.1 ml of freshly diluted enzyme-labeled antibody was added to each reaction well, incubated at 37°C for 0.5 to 1 hour; (4) washed: washed the sample with PBST for five times, 30 seconds each time; (5) added substrate solution to develop color; (6) stopped the reaction: added 0.05 ml of 2M sulfuric acid to each reaction well; (7) determined the OD value of each well at 450 nm with a microplate reader.

### 2.7. MPO and SOD Measurement

The MPO activity in colon tissue was determined using an MPO test kit (Nanjing Jiancheng Bio, China). The SOD activity in colon tissue was determined using a SOD test kit (Nanjing Jiancheng Bio, China).

### 2.8. Detection of IL-6, iNOS, and COX-2 in Colon Tissue by q-PCR

Real-time fluorescence quantitative polymerase chain reaction (qPCR) was chosen to measure mRNA expression in the colonic mucosa [22]. Total RNA was extracted from the colonic mucosa by the Trizol method; 1 *μ*g RNA was reverse transcribed in a 20 *μ*L reaction mixture in Prime Script RT Master Mix (TaKaRa, Japan). After reverse transcription, real-time PCR was performed with a real-time PCR instrument (ABI, USA). The primer sequences for each target mRNA are shown in Table 2. The reaction conditions were as follows: 95°C for 5 minutes, one cycle; alternate between 95°C for 5 s and 60°C for 30 s, 40 cycles.

### 2.9. Measurement of NF-*κ*B, i*κ*Ba, and p-i*κ*Ba Protein Expression in Colon Tissue by Western Blotting

The tissue was placed in a centrifuge tube with PBS buffer and then homogenized, centrifuged, lysed, and centrifuged to extract protein. The protein concentration was determined by a BCA kit (Beyotime, China). After polyacrylamide gel electrophoresis (Bio-RAD, USA), the membrane was transferred to a PVDF membrane (Millipore, USA) and immersed in blocking solution for 2 h. Primary antibody was added, and the membrane was incubated overnight at 4°C. After being washed, the membrane was incubated for 1 h at room temperature in the secondary antibody. After being washed again, the ECL Plus luminescence kit (Beyotime, China) was used for chemiluminescence development.

### 2.10. Statistical Analysis

All data were expressed as the mean ± standard deviation. SPSS 21.0 statistical software was used to compare the difference between groups by a one-way analysis of variance (ANOVA). P<0.05 was considered statistically significant.

## 3. Results

### 3.1. HLD Increased Body Weight and Weakened Disease Activity

**Figure 1:**
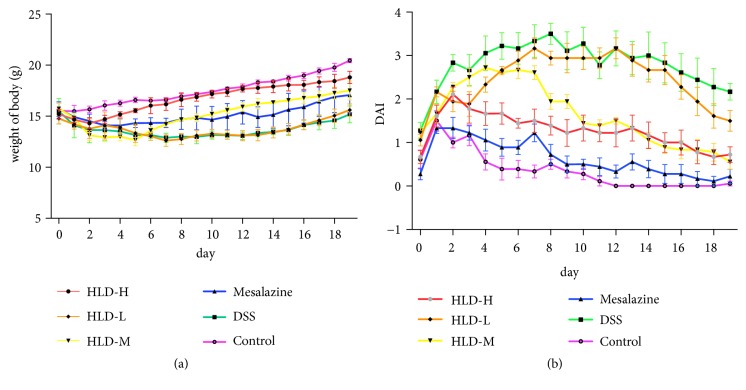
Effect of HLD on body weight (a) and evaluation of disease activity index (b). HLD-H and Mesalazine groups markedly decreased the DAI score and increased the body weight as compared to the DSS group (n=6).

No mice died in six groups during the experiment. HLD treatment significantly alleviated intestinal inflammation, increased body weight, and reduced the DAI score. As shown in Figure 1, the mice in the control group gradually gained weight through normal feeding. Compared with the Control group, the body weight of mice in the DSS group decreased gradually. The body weight of mice in the HLD-H, HLD-M, and Mesalazine groups increased gradually compared to the DSS group starting on the 2nd day after model establishment. The weight gain of HLD-H group was the most obvious. The DAI score of the Control group tended to be stable. Compared with the Control group, the DAI score of the DSS group increased gradually from the 2nd day after model establishment. Starting on the 2nd day after model establishment, the DAI scores of the HLD-H, HLD-M, and Mesalazine groups gradually decreased. Compared with the DSS group, the DAI scores of the Mesalazine and the HLD-H groups decreased significantly.

### 3.2. Comparison of Colon Macroscopic Lesion Scores

**Figure 2:**
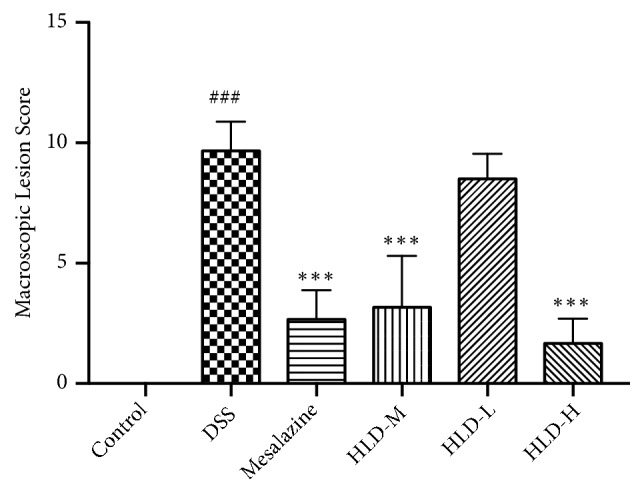
The data of colon macroscopic lesion score. Data are presented as the mean ± SD. ###P < 0.001 when compared to the control group; *∗∗∗*P < 0.001 when compared to the DSS group (n=6).

As shown in Figure 2, diarrhea, intestinal wall ulcers, and adhesions in the DSS group were more obvious than those in the Control group (P < 0.001), suggesting successful modeling. The colon macroscopic scores of the HLD-H, HLD-M, and Mesalazine groups were significantly lower than those of the DSS group (P<0.001), but there was no significant difference between the score of the HLD-L group (P>0.05) and that of the DSS group. The colon macroscopic scores of the HLD-H and HLD-M groups decreased compared to the Mesalazine group; however, the decrease was not significant (P>0.05).

### 3.3. HLD Alleviated Pathological Injury in Colon

**Figure 3:**
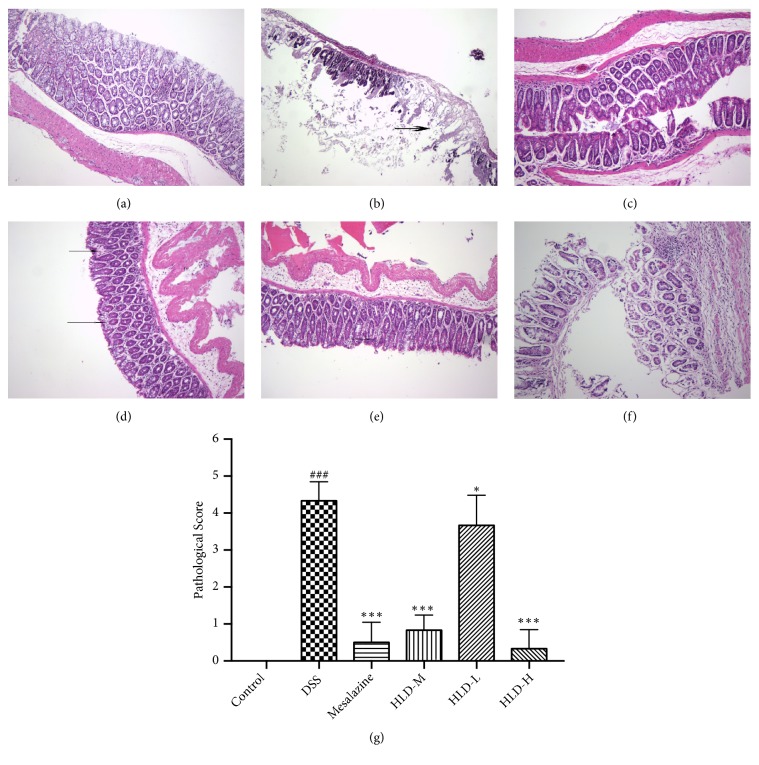
The HE staining (100×) of colon mucosa in control (a), DSS (b), Mesalazine (c), HLD-M (d), HLD-L (e), HLD-H (f), and the data of pathological score (g). (a) The colonic tissue from control mice showed intact mucosa without any histologic alteration. (b) DSS-induced colitis showed ulcer ( thick arrow). The colon wall structure of the Mesalazine (c) and the HLD-H (f) groups were approximately normal after treatment. (d) HLD-L treated mice do not fully recover from DSS-induced colitis, large amounts of inflammatory cells infiltrated in the colonic mucosa (arrow). (e) A few inflammatory cells infiltrated in the colonic mucosa of HLD-M group (arrow). Data are presented as the mean ± SD. ###P < 0.001 when compared to the control group; *∗∗∗*P < 0.001 when compared to the DSS group (n=6).

As shown in Figure 3, the colonic tissue from control mice showed normal mucosa without any histologic alteration. Compared with the Control group, the colonic mucosa of the DSS group was destroyed and the ulcer was formed. The inflammation of the intestinal wall was significantly more serious in the DSS group than in the Control group (P<0.001). Compared with the DSS group, the colon wall structures of the HLD-H and the Mesalazine groups were approximately normal after treatment (P<0.001), and a few inflammatory cells infiltrated in the colonic mucosa of HLD-M group. (P<0.001). However, although the intestinal wall inflammation was lighter in the HLD-L treatment group than the DSS group (P<0.05), it was still severe. Large amounts of inflammatory cells infiltrated in the colonic mucosa.

### 3.4. Effect of HLD on IL-6, TNF-*α*, and IL-1*β* in Colon Tissue

**Figure 4:**
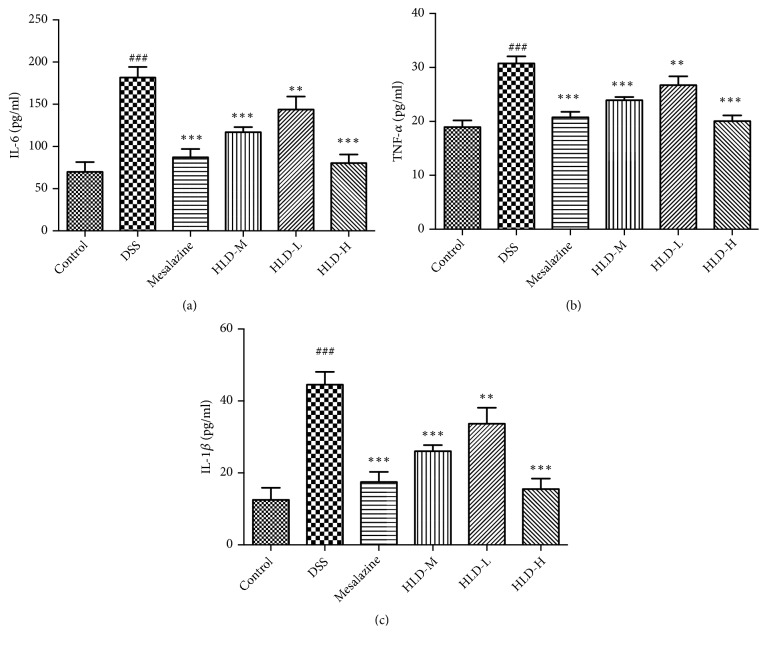
Effect of HLD on IL-6 (a), TNF-*α* (b), and IL-1*β* (c) in colon tissue. Data are presented as the mean ± SD. ###P < 0.001 when compared to the control group; *∗∗∗*P < 0.001, *∗∗*P < 0.01 when compared to the DSS group (n=6).

As shown in Figure 4, the levels of IL-6, TNF-*α*, and IL-1*β* in the DSS group were significantly higher than those in the Control group. However, compared with those in the DSS group, the levels of the abovementioned inflammatory cytokines were significantly decreased in the HLD-H, Mesalazine, and HLD-M groups after treatment. The decrease in inflammatory cytokine levels was especially pronounced in the HLD-H and Mesalazine groups, which were close to that of the Control group.

### 3.5. Effect of HLD on SOD and MPO Activity in Colon Tissue

**Figure 5:**
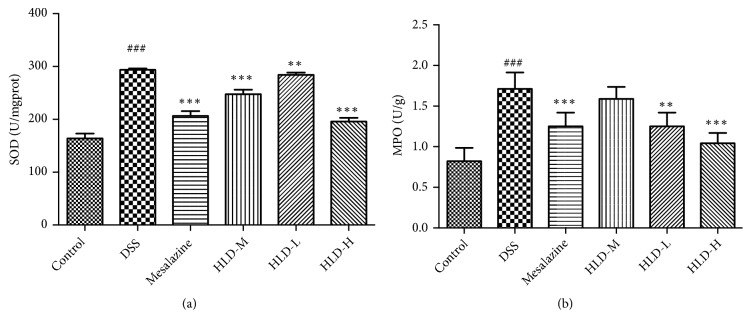
HLD decreased SOD (a) and MPO (b) activity in colon tissue. Data are presented as the mean ± SD. ###P < 0.001 when compared to the control group; *∗∗∗*P < 0.001, *∗∗*P < 0.01 when compared to the DSS group (n=6).

Compared to the Control group, the expression of SOD and MPO in the DSS group significantly increased. However, compared with the DSS group, the expression of SOD and MPO in the HLD-H and Mesalazine groups significantly decreased after treatment, as displayed in Figure 5. Interestingly, compared with the DSS group, the expression of SOD significantly decreased in the HLD-M group, while the expression of MPO remained same.

### 3.6. HLD Decreased the Expression of IL-6, iNOS, and COX-2 mRNA in Colon Tissue

**Figure 6:**
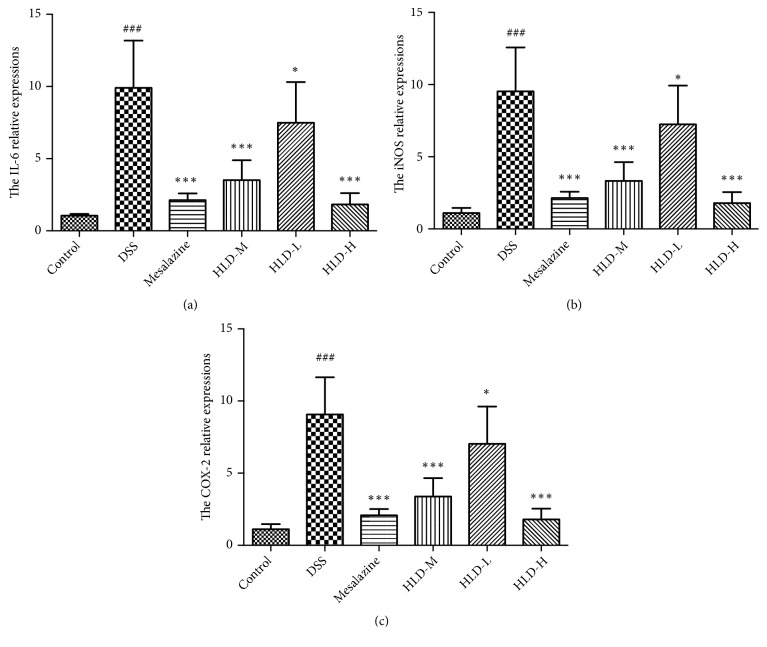
HLD decreased IL-6 (a), iNOS (b), and COX-2 (c) mRNA expressed in colon tissue. Data are presented as the mean ± SD. ###P < 0.001 when compared to the control group; *∗∗∗*P < 0.001, *∗*P < 0.05 when compared to the DSS group (n=6).

Compared with the Control group, the expression of IL-6, iNOS, and COX-2 mRNA significantly increased in the DSS group. However, compared with the DSS group, the mRNA expression of IL-6, iNOS, and COX-2 decreased significantly in the HLD-H, the Mesalazine, and the HLD-M groups, especially in the HLD-H and the Mesalazine groups, which were similar to the Control group. Compared with the DSS group, the mRNA expression of IL-6, iNOS, and COX-2 also decreased in the HLD-L group, but the decrease was not obvious, as shown in Figure 6.

### 3.7. HLD Reduced NF-*κ*B, i*κ*Ba, and p-i*κ*Ba Expression in Colon Tissue

**Figure 7:**
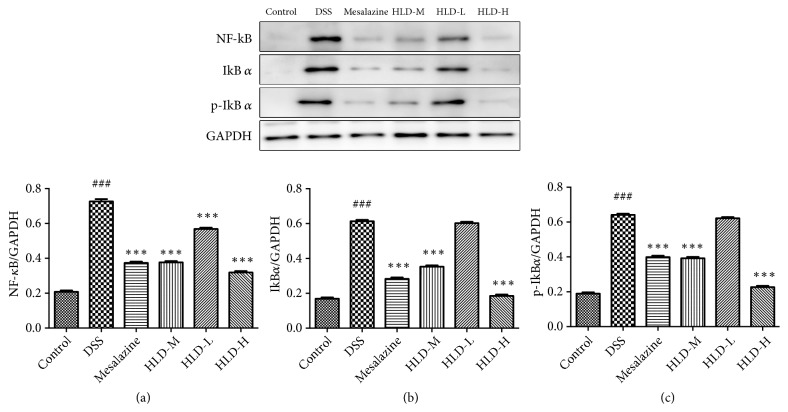
HLD decreased NF-*κ*B (a), IKB*α* (b), and p-IKB*α* (c) levels in colon mucosa. Data are presented as the mean ± SD. ###P < 0.001 versus the control group; *∗∗∗*P < 0.001, versus the DSS group (n=6).

Compared with the Control group, the protein expression of NF-*κ*B, i*κ*Ba, and p-i*κ*Ba significantly increased in the DSS group. However, compared with those in the DSS group, the NF-*κ*B, i*κ*Ba, and p-i*κ*Ba levels decreased significantly in the HLD-H, HLD-M, and Mesalazine groups, especially the HLD-H group. Unfortunately, compared with those in the DSS group, the levels of those inflammatory cytokines in the HLD-L group were not significantly reduced, as presented in Figure 7.

## 4. Discussion

So far, the main therapy goal of ulcerative colitis is to induce and maintain remission of the symptoms caused by intestinal inflammation and thereby improve the quality of life for these patients. DAI is a standard for scoring according to the patient's symptoms. It is one of the indicators for judging the degree of disease and evaluating the efficacy [31]. The DAI can well reflect whether the inflammation in UC patients is relieved [6]. This study showed that HLD enema effectively improved intestinal inflammation, reduced DAI, and decreased the expression of UC-related inflammatory cytokines in mouse UC induced by DSS. In particular, high-dose HLD treatment of DSS-induced mouse UC has comparable efficacy to Mesalazine, which is currently one of the main medications used to treat UC [32].

DSS-induced colitis is a reproducible model that morphologically and symptomatically resembles ulcerative colitis in humans [26]. DSS causes erosions with complete loss of surface epithelium because of its direct toxic effect on epithelial cells and significantly causes acute colitis. The morphological and macroscopic features of DSS-induced UC include hyperemia, ulceration, moderate to severe submucosal edema, and histopathological changes, which eventually manifest as bloody diarrhea [25, 33]. In the present study, oral DSS resulted in destruction of the mucosal barrier and colonic structure, and inflammatory cell infiltration. Diarrhea, intestinal wall ulcers, and adhesions in the DSS group were very obvious. Administration of HLD significantly alleviated the pathological injury of colon, reduced intestinal inflammation, increased body weight, and reduced DAI. Neutrophils contain a 140 kDa proteolytic enzyme called myeloperoxidase, which fights against bacteria [34]. SOD is an active substance derived from living organisms that can eliminate harmful substances produced during metabolism. Following the induction of colitis, MPO is released from neutrophils, oxidative stress occurs in the body, and the expression of SOD and MPO increases in colon tissue. However, these substances significantly decreased after administration of HLD.

5-ASA has been generally applied to IBD including UC treatment, but it has side effects. The number of IBD patients using herbs has increased to approximately 50%, but the clinical evidence and pharmacological mechanisms have not been clear, especially in UC treatment [6]. Thus, we focused on the NF-*κ*B pathway in UC treatment because the inhibitions of NF-*κ*B by 5ASA are clinically available mechanisms to ameliorate UC [6, 35].

Many studies have found that proinflammatory cytokines play an important role in the occurrence of UC, and the NF-*κ*B signaling pathway, which is a research hotspot, is crucial to the occurrence of IBD [8, 34]. TNF-*α*, which is a small molecule protein secreted by mononuclear macrophages, can improve the phagocytic ability of neutrophils and promote the adhesion of neutrophils to endothelial cells, thereby stimulating local inflammation [7]. Many studies found that TNF-*α* levels are elevated in murine models of IBD [36–39]. Inhibition of TNF-*α* secretion in IBD correspondingly reduced the severity of colitis [12, 40, 41]. NF-kB activity is stimulated by proinflammatory cytokines, such as TNF-a and IL-1, as well as by pathogen associated molecular patterns (PAMPs). IL-6 and IL-1*β* are closely related to the pathogenesis of IBD [36, 38, 41]. It was important that the further study attested such an assumption: the inhibitor of IL-1*β* reduced the levels of IL-1*β* and astrict the development of inflammation [6]. COX-2 is induced by various injurious factors and participates in the inflammatory response by catalyzing the synthesis of prostaglandins. Multiple stimulating factors increase the inducible expression of iNOS and lead to the synthesis of a large amount of NO, which mediates the inflammatory response. Many studies have confirmed that COX-2 and iNOS are associated with the pathogenesis of UC [42, 43]. In our experiment, treatment with HLD remarkably suppressed the enhanced tissue levels of IL-6, IL-1*β*, TNF-*α*, iNOS, and COX-2, which may account for the suppression of inflammatory infiltrates.

In the classical NF-*κ*B pathway, the (RelA / p65)/p50 heterodimer is maintained in the cytoplasm in an inactive state by the NF-*κ*B inhibitor (I*κ*Ba) family, which can mediate activation by regulating the large I*κ*B kinase complex consisting of the regulatory subunit I*κ*B kinase *γ* (I*κ*K*γ*), the catalytic subunit I*κ*B kinase *α* (I*κ*K*α*), and IkB kinase *β* (I*κ*K*β*)[1]. When the upstream kinase is activated, the I*κ*K complex phosphorylates I*κ*Ba, causing it to degrade and subsequently release the RelA/p50 heterodimer. The new RelA/p50 heterodimer is rapidly transferred to the nucleus to activate transcription of various inflammatory mediators such as COX-2, iNOS, TNF-*α*, IL-1*β*, and IL-6[7, 8]. Cytokines, oxidative stress, bacteria, viruses, and ischemia can stimulate and activate the NF-*κβ* pathway. Therefore, inhibition of NF-*κ*B signaling would be one of the therapeutic approaches to alleviate inflammation.

This study showed that the level of I*κ*Ba was significantly reduced in the cytoplasm of tissues after HLD treatment, and thus the phosphorylated i*κ*Ba (p-i*κ*Ba) level decreased. NF-kB was released and translocated into the nucleus, and the level was reduced accordingly. Finally, the levels of activated inflammatory mediators such as COX-2, iNOS, TNF-*α*, IL-1*β*, and IL-6 were reduced, and inflammation was relieved, which was in agreement with our hypothesis. Therefore, HLD mediated overproduction of IL-6, IL-1*β*, TNF-*α*, iNOS, and COX-2 could be correlated with that of NF-*κ*B activation. However, our experiments did not compare further the differences of NF-*κ*B protein expression between the cytoplasm and nucleus. If we can do as described in Pandurangan's study [44], the results will be perfect. Therefore, we will add more in-depth research in the further study of HLD in the treatment of DSS-induced mice UC.

Traditional Chinese medicine has a long history and has been widely used in Asia. An increasing number of animal experiments have confirmed that Chinese medicine monomers or compounds have good anti-inflammatory effects [14, 22, 45–47]. However, the aim of this research is to treat patients, and drugs are ultimately used on people. In clinical practice, we use Chinese medicine according to experience. However, this test suggests that we need to reevaluate the dose-effect relationship of traditional Chinese medicine administration in future clinical practice. In this experiment, we were surprised to find that doubling the usual dose of HLD in DSS-induced UC mice has a good effect and that the curative effect is comparable to that of Mesalazine. We need further clinical trials to evaluate the efficacy and safety of high-dose HLD enema in the treatment of UC patients.

## 5. Conclusions

This study showed that HLD has a good therapeutic effect on DSS-induced mouse UC and may involve the inhibition of the NF-*κ*B pathway. Additionally, the results suggested that high-dose HLD enema has a better effect on UC than the standard dose and has comparable efficacy to Mesalazine. At the same time, we suggest that we need to carry out clinical trials to evaluate the efficacy and safety of high-dose HLD enema in the treatment of UC and to provide guidelines for the rational clinical application of this drug.

## Figures and Tables

**Table 1:** DAI score.

score	weight loss (%)	stool consistency	bloody stools
0	None	Normal	None
1	1-5	Soft and shaped	Between
2	6-10	Loose	Fecal occult blood
3	11-15	Between	Between
4	>15	Diarrhea	Defecate hemorrhage

Note: DAI = (weight loss score + fecal shape score + bloody stool score) / 3, body weight decreased by 5% scored 1 point, and so on.

**Table 2:** Primer sequences for RT-PCR.

Gene	Primer sequences
IL-6	F: 5′GGCGGATCGGATGTTGTGAT3′
IL-6	R: 5′GGACCCCAGACAATCGGTTG3′
iNOS	F: 5′CAGGGAGAACAGTACATGAACAC3′
iNOS	R: 5′TTGGATACACTGCTACAGGGA3′
COX-2	F: 5′AACCCAGGGGATCGAGTGT3′
COX-2	R: 5′CGCAGCTCAGTGTTTGGGAT3′
GAPDH	F: 5′TGACCTCAACTACATGGTCTACA3′
GAPDH-	R: 5′CTTCCCATTCTCGGCCTTG3′

## Data Availability

All data generated or analysed in this study are included in this published article.
